# Circular RNA LIPH promotes pancreatic cancer glycolysis and progression through sponge miR‐769‐3p and interaction with GOLM1

**DOI:** 10.1002/ctm2.70003

**Published:** 2024-08-21

**Authors:** Yan Ma, Xiaomeng He, Yang Di, Wenyang Li, Lixiang Sun, Xin Zhang, Li Xu, Zhihui Bai, Zehuan Li, Lijun Cai, Huaqin Sun, Christopher Corpe, Jin Wang

**Affiliations:** ^1^ Central Laboratory Zhongshan Hospital (Xiamen), Fudan University Xiamen Fujian China; ^2^ Shanghai Public Health Clinical Center Fudan University Shanghai China; ^3^ Department of Pancreatic Surgery, Pancreatic Disease Institute Huashan Hospital, Shanghai Medical College, Fudan University Shanghai China; ^4^ Department of Physiology and Pathophysiology Hexi University School of Medicine Zhangye Gansu China; ^5^ Department of General Surgery Zhongshan Hospital, Fudan University Shanghai China; ^6^ Department of Nutritional Science King's College London London UK

Dear Editor,

Pancreatic cancer (PaCa) is a highly malignant tumour of the digestive system and is one of the major causes of cancer‐related death worldwide,^1^, ^2^, ^3^ and only approximately 10% of PaCa patients survive for 1 year after diagnosis.^4^, ^5^ Thus, investigations into sensitive and specific biomarkers for risk stratification are urgently needed for PaCa. Noncoding RNAs, including circRNAs, function as key ceRNAs (sponges) to regulate the expression of mRNAs, and their discovery greatly expanded the functional genetic information in carcinogenesis.^6^, ^7^, ^8^ CircRNAs are highly stable, are not easily digested by RNase, and can be detected in the saliva, blood and other body fluids of patients with cancer.^9^, ^10^

In this study, we revealed that a novel circular RNA (circLIPH/circ_0068398) was upregulated in pancreatic tumour tissue (Figure 1A). A high expression level of circLIPH was significantly correlated with tumour size, tumour stage and the percentage of Ki67‐positive tumours (*p* < .05; Table S1). circLIPH and lipase H (LIPH) expression levels were also significantly greater in most PaCa cells than in the hTERT‐immortalized epithelial (HPNE) control cells (Figure 1C,D). circLIPH is derived from exons 2 to 5 of the LIPH gene and has a length of 669 bp (Figure 1E); we amplified it from cDNA (not from gDNA) via divergent primers (Figure 1F). RNA fluorescence in situ hybridisation revealed circLIPH localisation primarily in the cytoplasm of PaCa cells (Figure 1G). We also demonstrated that circLIPH overexpression promoted the growth of BXPC‐3 and PANC‐1 cells via CCK‐8 assays (Figure S1E,F), whereas si‐circLIPH treatment significantly inhibited the proliferation of PaCa cells (Figure S1G,H). Colony formation assays (Figure S1I) revealed that circLIPH overexpression could effectively promote the growth of PaCa cells (Figure S1J) and that si‐circLIPH treatment inhibited cancer cell proliferation (Figure S1K). Wound healing (Figure S1L) and Transwell invasion assays (Figure S1O) demonstrated that circLIPH overexpression markedly enhanced the migration (Figure S1M) and invasion abilities (Figure S1P) of PaCa cells, whereas si‐circLIPH treatment impaired the migration (Figure S1N) and invasion (Figure S1Q) capabilities of PaCa cells. The protein levels of vimentin and Snail increased after circLIPH overexpression in PaCa cells, whereas the protein level of E‐cadherin decreased significantly (Figure S1R), which suggests that circLIPH may serve as an oncogene that facilitates cancer cell progression and promotes the epithelial–mesenchymal transition (EMT) of PaCa cells.

**FIGURE 1:**
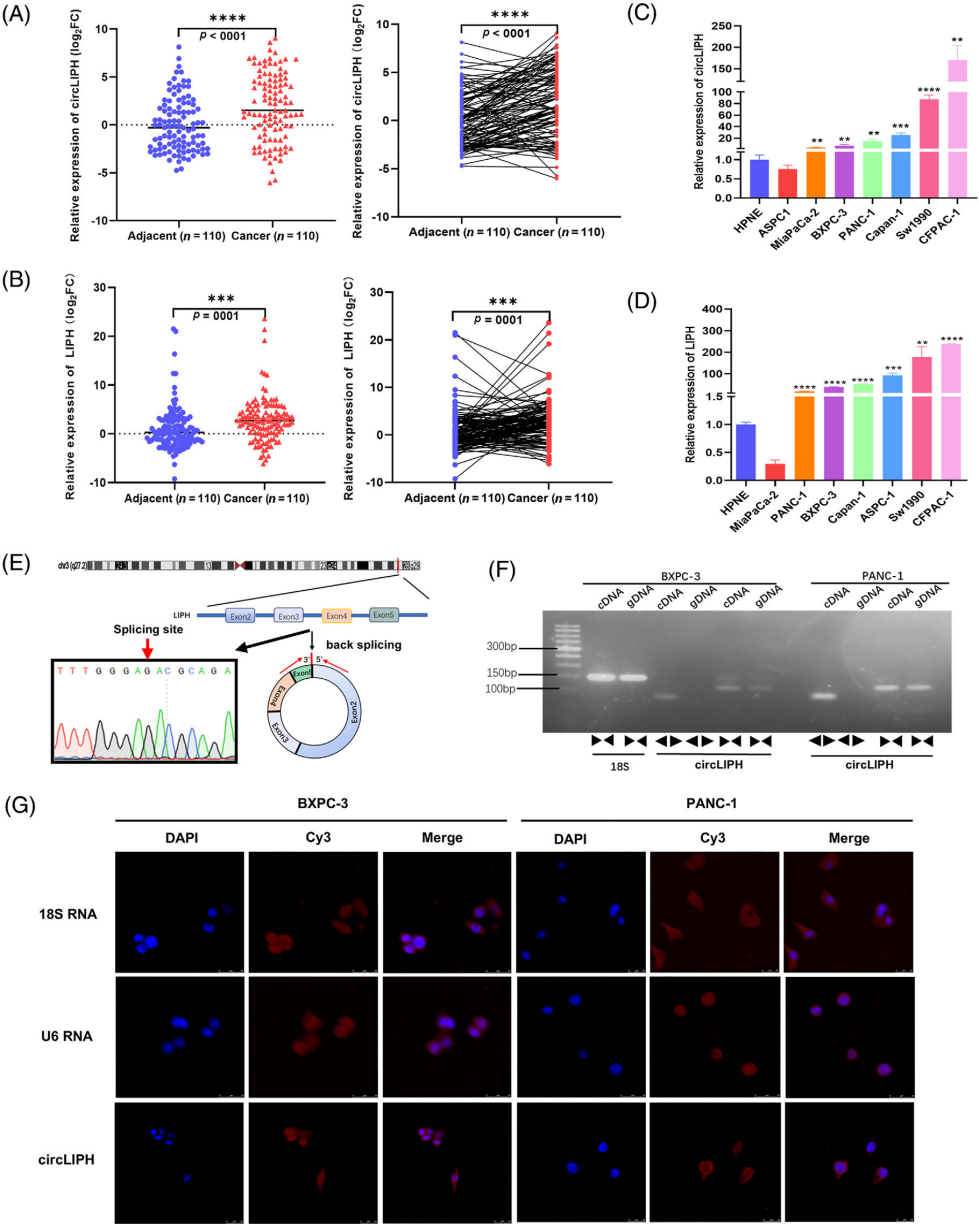
Identification and characteristics of circLIPH. The relative expression levels of circLIPH and LIPH were analysed in PaCa tissue samples (A, B) and pancreatic cells (C, D) via quantitative reverse transcription‐polymerase chain reaction (qRT‐PCR). Schematic illustration and Sanger sequencing showing the generation of circLIPH from exons 2 to 5 of the LIPH gene (E). PCR was used to amplify the circular and linear products to verify the presence of circLIPH in the BXPC‐3 and PANC‐1 cells (F). RNA fluorescence in situ hybridisation (FISH) was used to determine the location of circLIPH in BXPC‐3 and PANC‐1 cells (G). 18S and U6 were used as positive controls. Scale bar, 25 µm. All the data are shown as the means ± SDs.

To elucidate the molecular mechanism of circLIPH, seven candidate miRNAs were identified from the starBase, circBank and circInteractome databases (Figure 2A). Quantitative reverse transcription‐polymerase chain reaction (qRT‐PCR) analyses revealed that the level of miR‐769‐3p was decreased in BXPC‐3 and PANC‐1 cells after circLIPH was overexpressed (Figure 2B,C). The luciferase activity of wild‐type circLIPH was significantly inhibited in 293T cells transfected with miR‐769‐3p, but there was no significant change in the activity of the mutated circLIPH under the same conditions (Figure 2E). RNA immunoprecipitation (RIP) analyses confirmed the interaction between circLIPH and miR‐769‐3p and revealed that, compared with control immunoglobulin G (IgG), the AGO2 antibody strongly pulled down both circLIPH (Figure 2F,G) and miR‐769‐3p (Figure 2H,I) from the two PaCa cell lines. Next, to investigate the target genes of miR‐769‐3p, 16 potential gene targets of miR‐769‐3p that were identified by TargetScan were selected along with the upregulated genes in our PaCa SBC array (Figure 2J). The expression of the GOLM1 gene was negatively regulated by miR‐769‐3p overexpression (Figure 2O) or knockdown (Figure 2P) in both PaCa cell lines, and the expression level of the GOLM1 protein decreased after the overexpression of miR‐769‐3p (Figure 2Q). Cotransfection with miR‐769‐3p and the wild‐type 3′ the untranslated region (UTR) of GOLM‐1 significantly reduced luciferase activity but did not affect the mutated 3′UTR of GOLM1 (Figure 2S). GOLM1 was upregulated in PaCa tumour tissues and cancer cells (Figure 2T,U), and circLIPH enhanced the expression of GOLM1 (Figure 2 V,X); in contrast, transfection of si‐circLIPH repressed the expression of GOLM1 in both PaCa cell lines (Figure 2 W,X).

**FIGURE 2:**
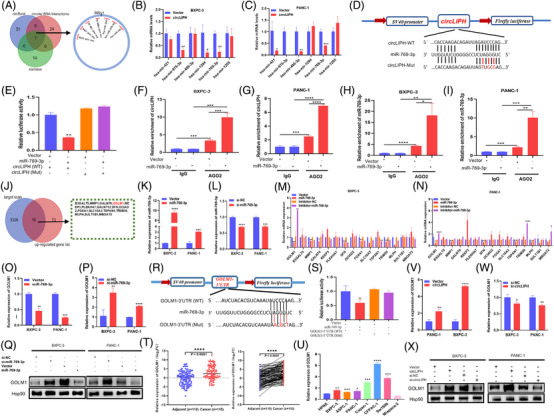
circLIPH functions as a miR‐769‐3p sponge and upregulates the expression of GOLM1 in PaCa. The potential target miRNAs of circLIPH were identified in the circBank, starBase and circInteractome databases, and shared potential targets are further prioritised via use of a Venn diagram (A). The expression levels of miRNAs in BXPC‐3 (B) and PANC‐1 cells (C) after circLIPH were overexpressed. Schematic illustration of dual‐luciferase reporter vectors expressing wild type (WT) or mutant (Mut) circLIPH (D). The luciferase activities in 293T cells cotransfected with circLIPH‐WT/Mut and miR‐769‐3p or the control vector (E). A RIP assay was conducted with anti‐Ago2 antibodies or IgG in pancreatic cancer cells after transfection with miR‐769‐3p (F–I). The potential target mRNAs of miR‐769‐3p identified via TargetScan and the upregulated genes identified via our SBC array were identified via a Venn diagram (J). Quantitative reverse transcription‐polymerase chain reaction (qRT‐PCR) was used to analyse the expression levels of miR‐769‐3p after overexpressing (K) or inhibiting miR‐769‐3p (L) in pancreatic cancer cells. Sixteen potential target mRNAs after overexpressing or inhibiting miR‐769‐3p in BXPC‐3 (M) and PANC‐1 cells (N). GOLM1 expression levels in BXPC‐3 and PANC‐1 cells after overexpressing (O) or inhibiting miR‐769‐3p (P) were analysed by qRT‐PCR and Western blotting (Q). Schematic illustration of dual‐luciferase reporter vectors expressing WT or Mut GOLM1 (R). Luciferase activity in 293T cells cotransfected with WT or Mut GOLM1 and miR‐769‐3p or the corresponding vector (S). GOLM1 mRNA expression levels in PaCa tissue samples (T) and pancreatic cells (U). GOLM1 expression levels after circLIPH overexpression or knockdown were analysed via qRT‐PCR (V, W) or Western blotting (X).

Furthermore, we found that the overexpression of miR‐769‐3p inhibited the growth and proliferation of PaCa cells via CCK‐8 assays (Figure 3A,B), whereas treatment with the miR‐769‐3p inhibitor had the opposite effects (Figure 3C,D). Colony formation assays (Figure 3E) revealed that overexpressing miR‐769‐3p inhibited the growth of PaCa cells (Figure 3F). However, treatment with the miR‐769‐3p inhibitor effectively promoted the growth of PaCa cells (Figure 3G). Wound healing (Figure 3H,I) and Transwell assays (Figure 3K,L) revealed that the migration and invasion abilities of PaCa cells were decreased in the miR‐769‐3p‐overexpressing group relative to those of the control group. In contrast, depletion of miR‐769‐3p increased PaCa cell migration (Figure 3J) and invasion (Figure 3M). Kyoto encyclopedia of genes and genomes (KEGG) analysis revealed that the target genes of miR‐769‐3p were enriched mainly in the mTOR signalling pathway (Figure 3N). The protein levels of p‐PI3K, p‐AKT, p‐mTOR, vimentin and Snail1 were dramatically increased by the overexpression of GOLM1, whereas si‐GOLM1 treatment significantly reduced the expression levels of these proteins (Figure 3O). The overexpression of miR‐769‐3p decreased not only the expression of GOLM1, vimentin and Snail1 but also the protein levels of p‐PI3K, p‐AKT and p‐mTOR, whereas the knockdown of miR‐769‐3p in PaCa cells increased the levels of mTOR‐related proteins (Figure 3P). Moreover, miR‐769‐3p treatment alleviated the activation of the mTOR pathway induced by circLIPH overexpression (Figure 3P). Our data suggest that miR‐769‐3p can inhibit EMT progression in PaCa cells via the mTOR signalling pathway. In addition, the extracellular acidification rate (ECAR) test revealed that circLIPH silencing decreased the production of protons by and decreased the extracellular acidification rates in BXPC‐3 and PANC‐1 cells (Figure 3Q,R). Thus, circLIPH may promote the progression of PaCa by enhancing glycolysis in cancer cells.

**FIGURE 3:**
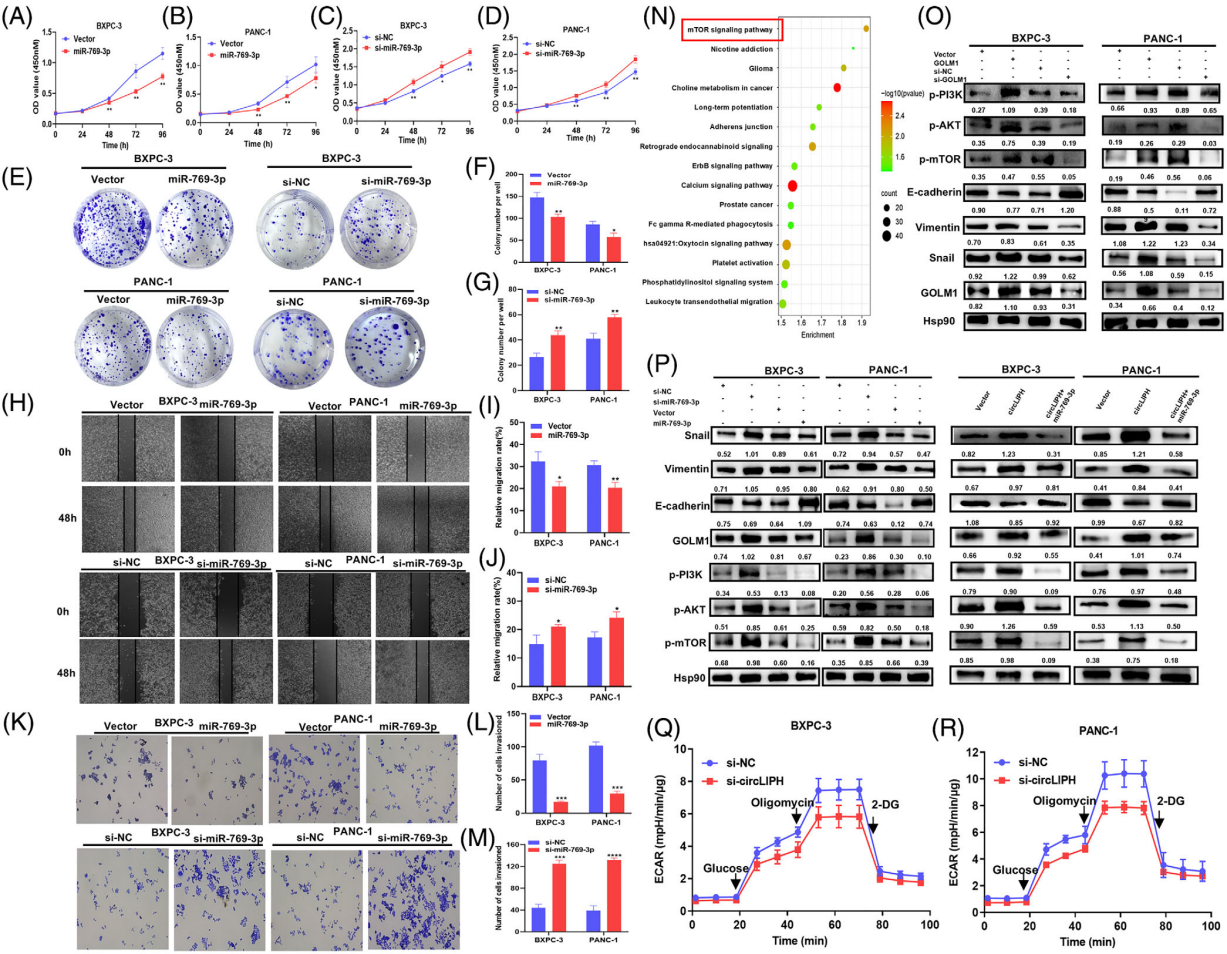
The inhibition of cell proliferation, migration and invasion by treatment with miR‐769‐3p was analysed via CCK‐8 (A–D), colony formation (E–G), wound healing (H–J) and Transwell (K–M) assays. KEGG pathway enrichment analysis of the target genes of miR‐769‐3p (N). The protein levels of p‐PI3K, p‐AKT, p‐mTOR, GOLM1, E‐cadherin, vimentin and Snail after overexpressing or inhibiting GOLM1 (O) or overexpressing circLIPH/cotransfecting circLIPH and miR‐769‐3p (P) were analysed via Western blotting. An ECAR assay was performed after circLIPH knockdown in BXPC‐3 (Q) and PANC‐1 cells (R).

Finally, we analysed the effects of an intratumour injection of si‐circLIPH in a xenograft PaCa tumour model (Figure 4A). The tumour volume was smaller in the si‐circLIPH‐treated group than in the control group (Figure 4B). In vivo imaging revealed that si‐circLIPH significantly inhibited tumour growth (Figure 4D–G). The qRT‐PCR results revealed that, relative to the control group and tumour tissues, the expression levels of circLIPH, GOLM1, vimentin and Snail1 were downregulated and those of miR‐769‐3p and E‐cadherin were upregulated in the circLIPH‐knockdown group (Figure 4H). Immunohistochemistry (IHC) analyses revealed that Snail was positively expressed in the cytoplasm and nucleus of tumour cells in the control group (Figure 4J) but weakly expressed in the tumour cells of the circLIPH‐knockdown group (Figure 4N), and vimentin was weakly positively expressed in the cytoplasm of tumour cells in the control group (Figure 4K) but almost absent in the cytoplasm of tumour cells in the circLIPH‐knockdown group (Figure 4O). On the other hand, the positive expression of E‐cadherin in the cytoplasm and cell membrane of tumour cells was verified in the circLIPH‐knockdown group (Figure 4P), whereas weak expression was detected in the cytoplasm and cell membrane of tumour cells in the control group (Figure 4L), which indicated that the inhibition of circLIPH suppressed the EMT process in vivo. Taken together, our results demonstrate that circLIPH may exert its oncogenic biological effects by activating the miR‐769‐3p/GOLM1/PI3K/AKT/mTOR axis (Figure 4Q) and that si‐circLIPH effectively represses circLIPH expression and suppress tumour growth via apoptosis in vivo.

**FIGURE 4:**
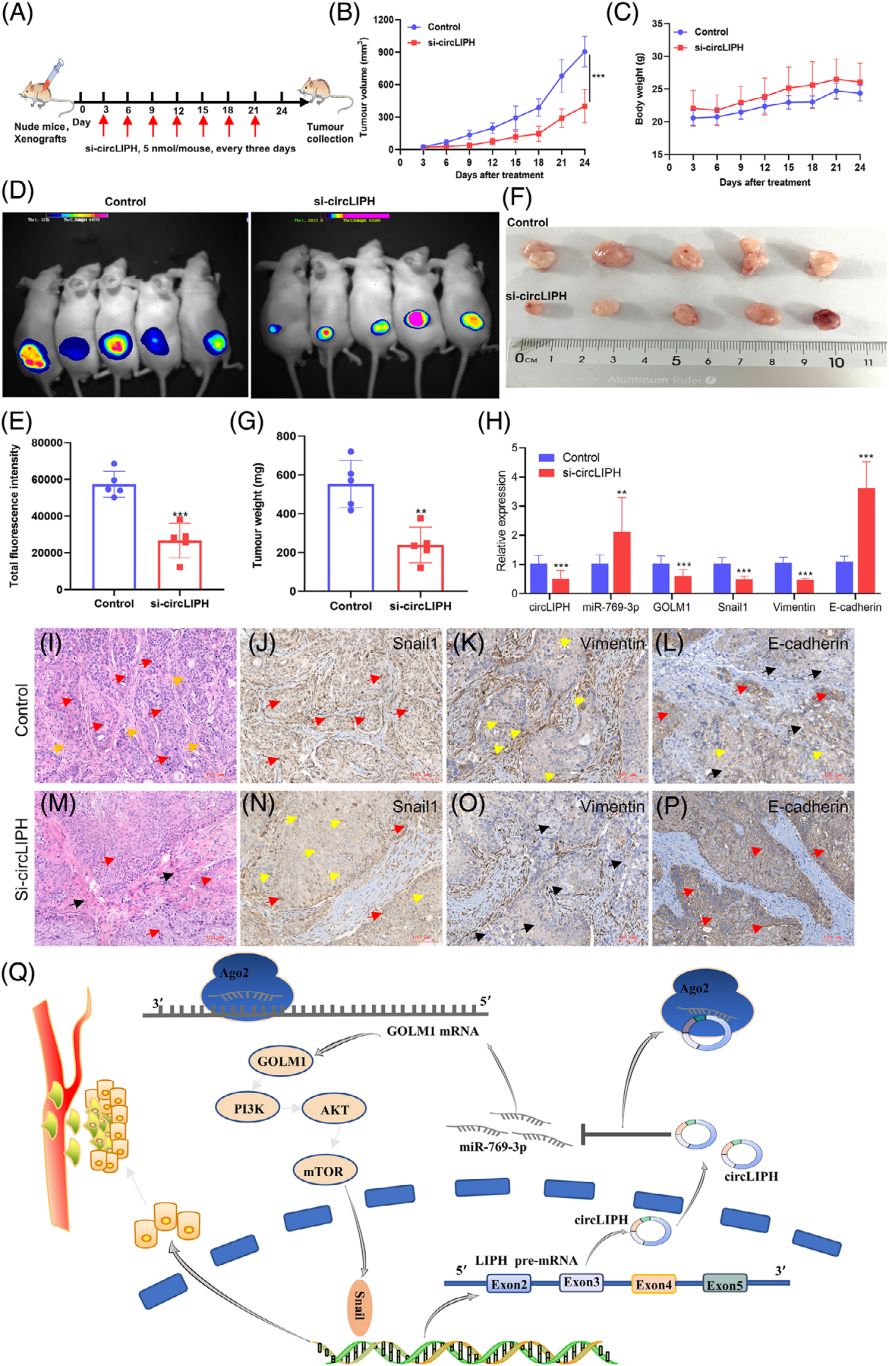
si‐circLIPH represses PaCa progression in vivo. Schematic illustration of the in vivo experiment showing that si‐circLIPH (5 nmol/mouse) or the control was injected into tumour‐bearing mice every 3 days (*n* = 5 for each group) (A). The tumour volume (B) and body weight (C) of the mice were measured every 3 days. Representative bioluminescence images of subcutaneous xenograft tumours (D, E). Representative images and weights of excised tumours (F, G). The mRNA expression levels of circLIPH, GOLM1, miR‐769‐3p, E‐cadherin, vimentin and Snail1were analysed in xenograft tumour tissues (H). Hematoxylin and eosin (HE) staining and IHC analyses of mouse xenograft tumour tissue samples from the intravenous injection of control (I–L) or si‐circLIPH (M–P) groups. HE staining (I, M: red arrows refer to tumour cells, yellow arrows refer to the vascular endothelium around the tumour and black arrows indicate the collagen fibrous tissue). IHC analyses of Snail1 (J, N), vimentin (K, O) and E‐cadherin (L, P) protein expression in mouse xenograft tumour tissue samples following the intravenous injection of control or si‐circLIPH (red arrows indicate positive expression, yellow arrows indicate weakly positive expression and black arrows indicate negative expression in tumour cells). Graphical abstract of circLIPH activating the miR‐769‐3p/GOLM1/PI3K/AKT/mTOR axis in PaCa cells (Q).

## AUTHOR CONTRIBUTIONS

Jin Wang conceived and designed the experiments, secured funding for the project, and supervised the research; Yan Ma conducted the experiments; Yang Di collected the clinical tissues; Xiaomeng He and Wenyang Li analyzed the data; Lixiang Sun, Xin Zhang, Li Xu, Zhihui Bai, Zehuan Li, Lijun Cai and Huaqin Sun assisted with the research and critically reviewed the paper; Jin Wang, Yan Ma, and Christopher Corpe edited the manuscript. All the authors have read and approved the final manuscript.

## CONFLICT OF INTEREST STATEMENT

The authors declare no conflicts of interest.

## ETHICS STATEMENT

Patient tumour tissue sample collection was approved by the Ethics Committee of Zhongshan Hospital (Xiamen) (B2024‐018) and Shanghai Public Health Clinical Center (2020‐S027‐02), Fudan University, China. All animal experiments were carried out following NIH Guidelines for the Care and Use of Laboratory Animals and approved by the Animal Care Committee of Shanghai Public Health Clinical Center (2020‐A033‐01).

## Supporting information

Supporting Information
