# Supplementary material for: Circular RNA LIPH promotes pancreatic cancer glycolysis and progression through sponge miR‐769‐3p and interaction with GOLM1
Source: Clin Transl Med. 2024 Aug 21;14(8):e70003. doi: 10.1002/ctm2.70003 (PMC11337905; doi:10.1002/ctm2.70003)

**Supplemental Information**

**Circular** **RNA LIPH promotes pancreatic cancer glycolysis and progression through sponge miR-769-3p and interaction with GOLM1**

Yan Ma^1, 2, #^, Xiaomeng He^2, #^, Yang Di^3, #^, Wenyang Li^4, #^, Lixiang Sun^1^, Xin Zhang^1^, Li Xu^2^, Zhihui Bai^1^, Zehuan Li^5^, Lijun Cai^1^, Huaqin Sun^1^, Christopher Corpe^6^, Jin Wang^1, 2,*^

^1^Central Laboratory, Zhongshan Hospital (Xiamen), Fudan University, Xiamen 361015, Fujan, China; ^2^Shanghai Public Health Clinical Center, Fudan University, 2901 Caolang Road, Jinshan District, Shanghai, China; ^3^Department of Pancreatic Surgery, Pancreatic Disease Institute, Huashan Hospital, Shanghai Medical College, Fudan University, Shanghai, China; ^4^Department of Physiology and Pathophysiology, Hexi University School of Medicine, Zhangye, Gansu 734000, China; ^5^Department of General Surgery, Zhongshan Hospital, Fudan University, 180 Fenglin Road, Xuhui District, Shanghai 200032, China; ^6^King's College London, London, Department of Nutritional Science, 150 Stamford Street, Waterloo, London, SE19NH, United Kingdom.

**Running Title**: circLIPH promotes PaCa progression

**Keywords:** Pancreatic cancer; circLIPH, miR-769-3p, GOLM1, mTOR

***Correspondence should be addressed to:**

Jin Wang, Ph.D.

Central Laboratory,

Zhongshan Hospital (Xiamen),

Fudan University,

Xiamen 361015, China

Ph: 86-17701869068;

Email: wang.jin@zsxmhospital.com

^ORCID ID: orcid.org/0000-0002-0062-2489^

^#^ These authors contributed equally to this work

**1. Supplementary Table S1.** Correlation analyses of clinicopathological characteristics with the expression levels of circLIPH in 110 patients with PaCa.

| **Clinicopathologic feature** | **All cases** | **circLIPH** | | ***P* value** |
| --- | --- | --- | --- | --- |
|  |  | **High expression** | **Low expression** |  |
| **Age** |  |  |  |  |
| ≤ 60 | 37 | 19 | 18 | > 0.999 |
| > 60 | 73 | 36 | 37 |  |
| **Sex** |  |  |  |  |
| Male | 68 | 32 | 36 | 0.556 |
| Female | 42 | 23 | 19 |  |
| **Tumour diameter (cm)** |  |  |  |  |
| ≤ 4 | 73 | 26 | 47 | **< 0.0001****** |
| > 4 | 34 | 28 | 6 |  |
| **Tumour stage** |  |  |  |  |
| Ⅰ/Ⅱ | 49 | 12 | 37 | **< 0.0001****** |
| Ⅲ/Ⅳ | 48 | 36 | 12 |  |
| **Ki67 positive rate (%)** |  |  |  |  |
| ≤ 10 | 25 | 8 | 17 | **0.023*** |
| > 10 | 73 | 43 | 30 |  |
| **CA19-9 (U/ml)** |  |  |  |  |
| ≤ 37 | 28 | 11 | 17 | 0.190 |
| > 37 | 80 | 44 | 36 |  |
| **CA125 (U/ml)** |  |  |  |  |
| ≤ 35 | 86 | 44 | 42 | 0.439 |
| > 35 | 18 | 7 | 11 |  |

Fisher's exact test. * represents *p* < 0.05, ** represents *p* < 0.01, *** represents *p* < 0.001, and **** means *p* < 0.0001.

**2. Supplementary Figure S1.** circLIPH promotes the proliferation, migration, and invasion of PaCa cells. The relative expression of circLIPH in pancreatic cancer cells after transfection with the circLIPH overexpression vector (**A, B**) or three si-circLIPH vectors (**C, D**) was analysed by qRT‒PCR. The ability of circLIPH to promote cell proliferation, migration, and invasion was assessed by CCK-8 (**C–H**), colony formation (**I–K**), wound healing (**L–N**), and Transwell (**O–Q**) assays. The protein levels of E-cadherin, Vimentin and Snail were analysed in PaCa cells after overexpressing or knocking down circLIPH by western blotting (**R**).


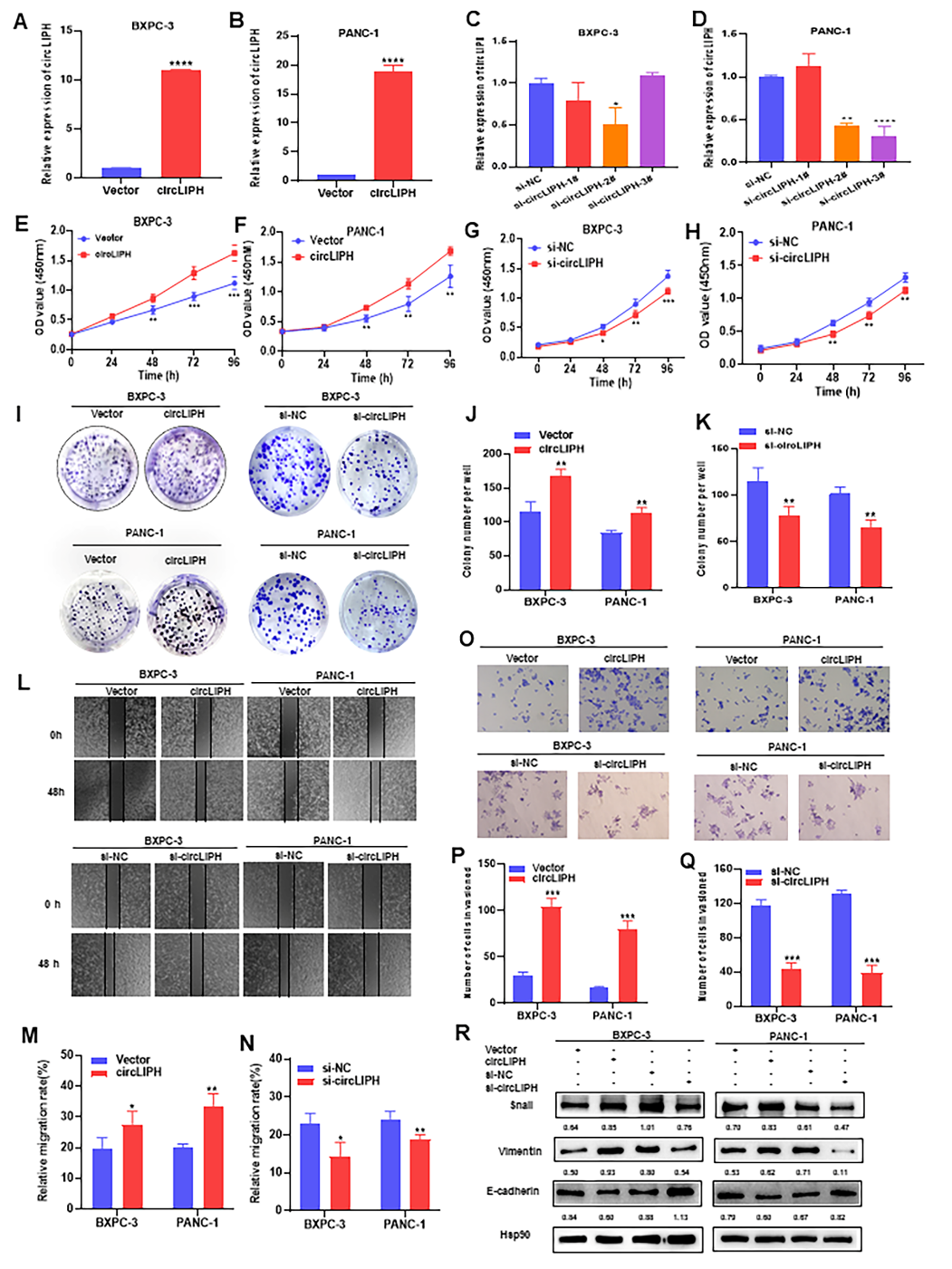

Supplement: Supplementary file 1 — Supporting Information [file CTM2-14-e70003-s001.docx]
